# Biomechanical Responses of Neonatal Brachial Plexus to Mechanical Stretch

**DOI:** 10.1055/s-0038-1669405

**Published:** 2018-09-03

**Authors:** Anita Singh, Shania Shaji, Maria Delivoria-Papadopoulos, Sriram Balasubramanian

**Affiliations:** 1 School of Engineering, Widener University, Chester, Pennsylvania, United States; 2 Department of Pediatrics, Drexel University College of Medicine, Philadelphia, Pennsylvania, United States; 3 School of Biomedical Engineering, Sciences and Health Systems, Drexel University, Philadelphia, Pennsylvania, United States

**Keywords:** Neonatal Brachial Plexus, injury mechanism, biomechanical properties, stretch rates

## Abstract

This study investigated the biomechanical responses of neonatal piglet brachial plexus (BP) segments—root/trunk, chord, and nerve at two different rates, 0.01 mm/second (quasistatic) and 10 mm/second (dynamic)—and compared their response to another peripheral nerve (tibial). Comparisons of mechanical responses at two different rates reported a significantly higher maximum load, maximum stress, and Young's modulus (E) values when subjected to dynamic rate. Among various BP segments, maximum stress was significantly higher in the nerve segments, followed by chord and then the root/trunk segments except no differences between chord and root/trunk segments at quasistatic rate. E values exhibited similar behavior except no differences between the chord and root/trunk segments at both rates and no differences between chord and nerve segments at quasistatic rate. No differences were observed in the strain values. When compared with the tibial nerve, only mechanical properties of BP nerves were similar to the tibial nerve. Mechanical stresses and E values reported in BP root/trunk and chord segments were significantly lower than tibial nerve at both rates. When comparing the failure pattern, at quasistatic rate, necking was observed at maximum load, before a complete rupture occurred. At dynamic rate, partial rupture at maximum load, followed by a full rupture, was observed. Occurrence of the rate-dependent failure phenomenon was highest in the root/trunk segments followed by chord and nerve segments. Differences in the maximum stress, E values, and failure pattern of BP segments confirm variability in their anatomical structure and warrant future histological studies to better understand their stretch responses.

## Introduction

Despite improvements in obstetrical care, neonatal brachial plexus palsy (NBPP) continues to occur in 1.1 to 2.2 per 1,000 births and remains a challenge for the affected families and treating physicians. 1 A major risk factor for NBPP is shoulder dystocia, where the fetal shoulder is impacted against the maternal pubic bone during vaginal birth resulting in stretching of the brachial plexus (BP) nerve or avulsion of its roots. 2 3 4 5 6 7 Because in vivo measurements of the exerted forces, the fetal shoulder deformation, and the resulting response of BP are technically difficult, computational and physical models are used to simulate these events. 4 5 7 While the data obtained from these models help demonstrate the effects of forces on the BP, they are based on the nonlinear mechanical properties of the rabbit tibial nerve. 8 BP is a complex structure formed by the ventral rami of the C5–T1 nerve roots followed by trunk, chord, and nerve segments. So while these data are currently the best available, using the mechanical properties of another peripheral nerve might not fully simulate the responses from the entire neonatal BP tissue.

This study aims to provide a detailed understanding of the biomechanical properties of neonatal BP, using a piglet animal model, in response to tensile loading by testing various segments of the BP complex (i.e., root/trunk, chord, and nerve) to quasistatic stretch rate (0.01 mm/second) and dynamic stretch rate (10 mm/second), and comparing the biomechanical responses of various segments of the BP to the tibial nerve under the two loading conditions.

## Methods and Materials

### Tissue Harvest

**Fig. 1:**
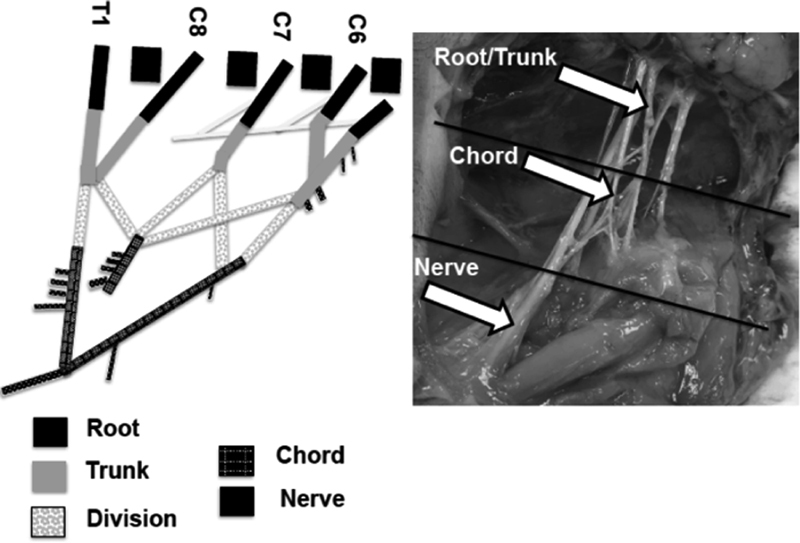
Brachial plexus segments.

A total of 114 immediately postpartum BP segments (root/trunk, chord, and nerve) and 22 tibial nerves from 11 normal neonatal piglets (3–5 days old) were used in this in vitro study. Using an axillary approach, with the animals in a supine position, BP complex was exposed on both sides of the spine. The lower three cervical (C6–8) and first thoracic (T1) spinal vertebral foramens were then identified and the plexus was carefully examined to locate the bifurcations of the divisions (M shape) as shown in Fig. 1 . BP segments above these bifurcations toward the spine and in the supraclavicular part of the plexus were labeled as root/trunk and those below these bifurcations were labeled as chord followed by nerve. The lateral chord was traced from ventral division of the upper and middle trunk, while the ventral division of the lower trunk formed the medial chord. When the chords bifurcated laterally closer to the arm, nerves including ulnar, median, and radial were identified and harvested. Tibial nerves from these animals were also harvested using a lateral approach. These freshly harvested tissues were preserved in 1% bovine serum albumin until testing, which was performed within 2 hours from harvesting.

### Mechanical Test Setup

**Fig. 2:**
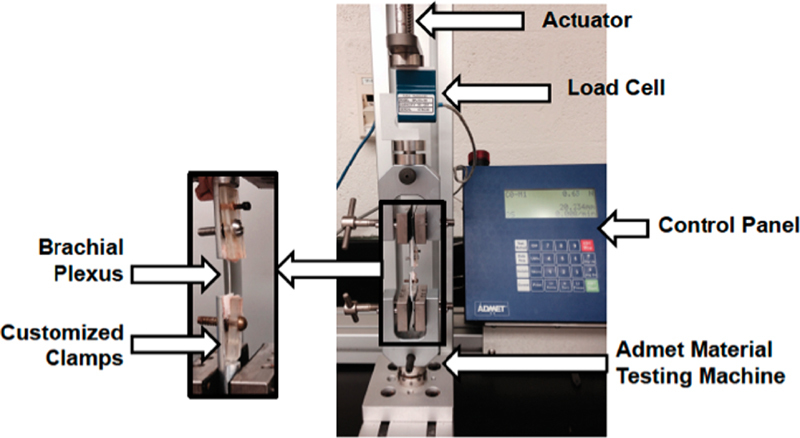
Biomechanical testing machine.

An ADMET material testing machine (eXpert 7600, ADMET Inc., Norwood, Massachusetts, United States) was used to stretch the BP segments and the tibial nerves ( Fig. 2 ). Each tissue was anchored to the testing setup by specially designed and fabricated clamps ( Fig. 2 ). Detailed design of the clamp has been reported previously. 9 The design of the clamp allowed for clamping each segment firmly between the padded Plexiglass and flat surface of the cylinder. The padded side facing the segment minimized the stress concentration at the clamping site. One clamp was attached to the fixed end of the machine and other end to the actuator and 200 N load cell of the testing machine.

### Camera System Setup

A high-speed video camera (Basler acA640–120uc camera (Basler, Exton, Pennsylvania, United States), which collected data at 120 fps was positioned in front of the material testing machine to capture the images of the tissue during the pull.

### Testing Procedures

Bilateral BP segments and tibial nerves were divided into two groups (Group A and Group B). A minimum of eight samples were tested for each BP segment and tibial nerve in the two groups. Specimens in Group A were subjected to a stretch rate of 0.01 mm/second (quasistatic) and those in Group B were subjected to a stretch rate of 10 mm/second (dynamic; Table 1 ). A digital microscopic was used to obtain images of harvested BP segments and tibial nerve before stretch (5X; Digital VHX Microscope, Elmwood Park, New Jersey, United States). A 2-mm scale (Leitz, Ernst-Leitz-Wetzlar GmbH, Oberkochen, Germany) at the same magnification was used to measure the tissue diameter.

**Table 1:** Summary (mean ± SEM) of maximum load (N), maximum stress (MPa), strain at maximum stress, and E (MPa) values of brachial plexus (BP) complex

	BP complex		
Rates	0.01 mm/s	10 mm/s	*p*
*N*	54	50	
Diameter (mm)	1.93 ± 0.14	1.88 ± 0.13	
Maximum load (N)	1.83 ± 0.30	3.52 ± 0.42	0.002
Maximum stress (MPa)	0.56 ± 0.07	1.15 ± 0.15	0.001
Strain	0.32 ± 0.03	0.32 ± 0.02	
E value (MPa)	2.87 ± 0.32	5.27 ± 0.69	0.003

The two clamps were initially set at a distance of 10 to 20 mm (depending on the initial length of the tissue) and the testing sample was then clamped with no initial tension prior to stretch. Stretch rates were controlled by built-in GaugeSafe software (ADMET Inc.), which pulled the clamped sample at the assigned rate (0.01 or 10 mm/second) until complete failure occurred. During this tensile test, time, load, and displacement data were acquired at a sampling rate of 25 Hz for quasistatic and 1,000 Hz for dynamic stretch rates. After the completion of the experiment, the failure site was recorded (at or closer to actuator side, at or closer to stationary side, or mid-length of the sample). Finally, the clamps were checked for the presence of tissue. No tissue in the clamps implied that the tissue had completely slipped, and the results of those experiments were discarded.

### Data Analysis

Load readings were converted to nominal stresses (load/original cross-sectional area of the sample). Displacement data were used to calculate the tensile strain (strain = (L _f_ − L _i_ )/L _i_ , where L _i_ is the initial length and L _f_ is the final length) exerted during the pull. The load–displacement and stress–strain curves were plotted and the maximum load, maximum stress, strain at the point of maximum stress, and Young's modulus (E values; the slope of stress–strain curve after the toe region and below the proportional limit) were determined. The camera data were used to track changes in structural integrity of the tested segment. As the load, displacement, and image data were recorded synchronously, the relationships between the three datasets could be characterized.

### Statistical Analysis

Statistical analysis was performed using SPSS software (Chicago, Illinois, United States). Values were expressed as mean ± standard error of mean (mean ± SEM). Maximum load, maximum stress, strain at maximum stress, and E values were compared using two-way ANOVA with two independent variables—stretch rate and tissue samples (BP segments and tibial nerve). Subsequent pairwise comparisons were conducted by independent *t* -tests on the stretch rate, and by one-way ANOVA (post hoc: Bonferroni) on the tissue samples in various categories. A *p* value of less than 0.05 was considered significant.

## Results

Out of the 114 BP segments, 57 segments were subjected to the quasistatic stretch rate of 0.01 mm/second (Group A) and 57 were subjected to the dynamic stretch rate of 10 mm/second (Group B). 96.5% (54/57) of the BP segments in Group A and 93.0% (50/57) in Group B did not slip at the clamps. 91% (10/11) of the tibial nerve tissue in Group A and 72.7% (8/11) in Group B did not slip at the clamps. Samples that indicated any slip at the clamping surfaces were not considered for data analysis. Table 1 summarizes the mechanical responses of the BP complex. Table 2 summarizes the total number of BP segments obtained from the BP complex and tibial nerve that were included in the data analysis. Failure was observed over the entire length of the tissue. In 72% (57% in quasistatic and 43% in dynamic) of the observed cases, the rupture occurred along the length of the segment closer to the fixed end of the testing machine, whereas in the remaining 28% (43% in quasistatic and 57% in dynamic) of the cases the rupture was observed closer to the actuator/moving end of the machine.

**Table 2:** Summary (mean ± SEM) of maximum load (N), maximum stress (MPa), strain at maximum stress, and E (MPa) values of various brachial plexus segments and peripheral tibial nerve tissue when subjected to two stretch rates (0.01 and 10 mm/s)

	BP trunks		BP chords		BP nerves		Tibial nerve	
Rates	0.01 mm/s	10 mm/s	0.01 mm/s	10 mm/s	0.01 mm/s	10 mm/s	0.01 mm/s	10 mm/s
*N*	32	25	14	13	8	12	10	8
Maximum load (N)	1.08 ± 0.10	2.12 ± 0.28 a	1.49 ± 0.30	4.77 ± 0.72 a	2.79 ± 0.71	4.97 ± 1.84	2.53 ± 0.70	4.81 ± 1.01
Maximum stress (MPa)	0.20 ± 0.02	0.45 ± 0.04 a	0.46 ± 0.02	1.31 ± 0.08 a	0.98 ± 0.10	3.51 ± 0.44 a	1.17 ± 0.26	3.07 ± 0.41 a
Strain	0.24 ± 0.04	0.34 ± 0.05	0.37 ± 0.07	0.32 ± 0.03	0.37 ± 0.06	0.29 ± 0.05	0.42 ± 0.08	0.34 ± 0.05
E value (MPa)	1.48 ± 0.19	2.02 ± 0.21 a	2.41 ± 0.40	6.39 ± 0.67 a	4.51 ± 0.53	14.87 ± 1.59 a	5.27 ± 1.35	14.83 ± 1.03 a

Note: Numbers of samples tested per segments are also provided.

a Significant differences between the two rates (0.01 and 10 mm/s).

### Effect of Stretch Rates on Mechanical Properties

**Fig. 3:**
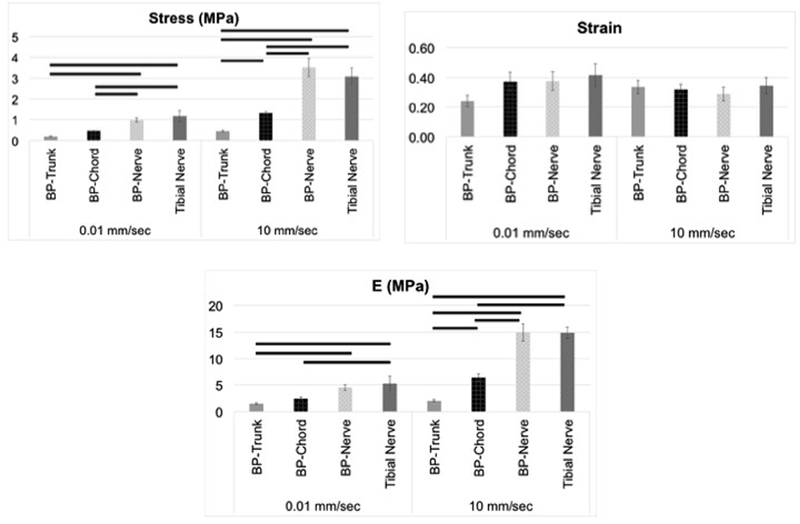
Mean ± standard error of mean (SEM) values of maximum stress, strain at maximum stress, and E values observed at various segments of the brachial plexus (BP) and the tibial nerve, when subjected to two different stretch rates. SEM values are shown as error bars. Significant differences ( *p* < 0.05) in the values among various segments of the BP are indicated using a dark solid line above the bars.

**Fig. 4:**
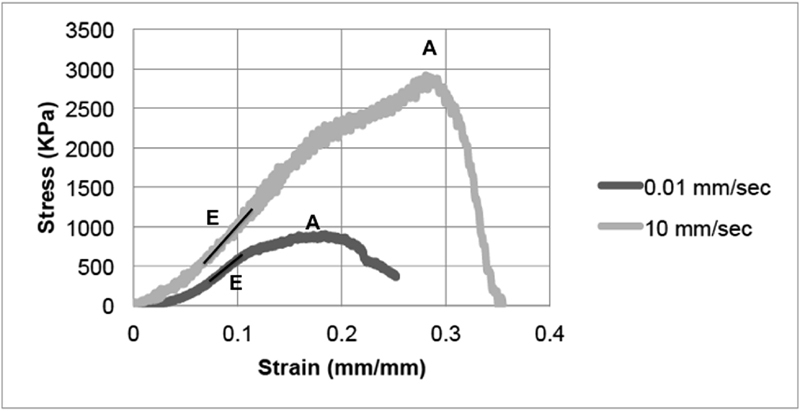
Comparison of the mechanical behavior of trunk segment when subjected to two different stretch rates. A: Point at which maximum stress and corresponding strain were obtained. E: Region where modulus of elasticity was calculated.

Differences in the mechanical behavior of the BP segments and tibial nerve were observed between the two different stretch rates ( Figs. 3 and 4 , Tables 1 and 2 ) such that the maximum load, maximum stress, and E values were significantly higher in Group B than in Group A ( *p* < 0.05, independent *t* -tests, Table 1 ). There was no statistical difference in the strain values between the two groups.

### Mechanical Properties of Brachial Plexus Segments

When comparing mechanical properties among various segments, we observed significantly higher maximum stresses in the BP nerve than in the chord followed by those observed in the root/trunk segments ( Table 2 and Fig. 3 ) at both rates, except that no significant differences existed between chord and root/trunk segments at quasistatic rate. When comparing E values, at both stretch rates, similar responses were observed, except that E values were not significantly different between chord and nerve segments at the quasistatic stretch rate. Also, there were no significant differences in the strain values among various BP segments at both rates.

### Comparing Mechanical Properties of Brachial Plexus Segments and Tibial Nerve

When comparing mechanical properties between various BP segments and the tibial nerve, BP nerve was not significantly different from the tibial nerve at both rates ( Fig. 3 ). When comparing other BP segments, significantly higher maximum stresses and E values were reported in tibial nerves than in root/trunk and chord segments of the BP at both rates. There were no significant differences in the strain values among various BP segments and tibial nerve at both rates.

### Failure Pattern

**Fig. 5:**
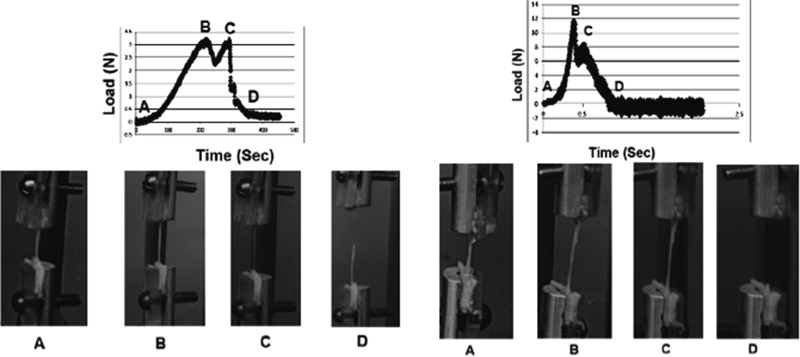
Load–time graph and corresponding images of the brachial plexus segments subjected to quasistatic (left) and dynamic (right) stretch rate. On the graph: Point A is the toe region, Point B is the maximum load, Point C is the rupture load, and Point D is the complete rupture of the tissue.

**Fig. 6:**
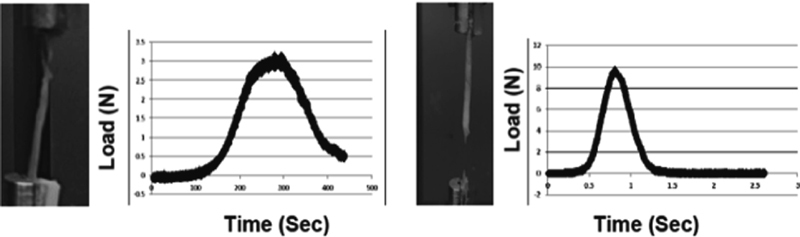
Images and load–time graph of segments where a sudden failure of the segment was observed in both groups (quasistatic: left, dynamic: right) and the point of failure typically occurred at the maximum load, beyond the proportional limit.

When comparing the failure patterns using load and image data, different failure modes were observed for the two groups. In Group A (0.01 mm/second), slight necking in the segment was found at the maximum load as shown in Fig. 5 (71% in root/trunk, 22% in chord, 14% in BP nerve, and 12% in tibial nerve). Necking was defined as a decrease in the segment diameter with an intact outer structure. On the other hand, in Group B (10 mm/second), with the increasing strain, the segment became progressively thinner over the entire length and was partially torn at the proportional limit and completely at the rupture load as shown in Fig. 5 (90% in root/trunk, 46% in chord, 30% in BP nerve, and 32% in tibial nerve). In these cases, the maximum load attained after the proportional limit ( Fig. 5 , Point B) did not correspond to the complete failure of the tissue. After the proportional limit, the maximum load dropped followed by a slight increase in the load until a complete rupture of the tissue occurred ( Fig. 5 , Point C) indicating that the maximum load sustained by the tissue was not the failure load. In the remaining cases as shown in Fig. 6 , a sudden failure of the segment was observed in both groups and the point of tissue failure typically occurred at the maximum load, just beyond the proportional limit.

## Discussion

NBPP occurs during birth when forces, both exogenous (clinician applied forces) and endogenous (maternal forces), stretch the BP beyond its elastic limit. The effect of these forces on the BP is directly related to magnitude, loading rate, surrounding tissue properties, and how the overall applied force is transmitted to the BP itself (including the alignment of the force vector with the axis of the BP bundle or its segments). Available literature reporting the effects of these factors on the BP tissue exhibits a wide discrepancy ( Table 3 ). Tissue processing (e.g., fixed, unfixed tissue), methodological differences in measuring elongation, and differences in species contribute to variations in the results. Furthermore, no study used fresh tissue or reported its response at various loading rates in neonatal BP tissue. Having mechanical data from human neonates would be ideal to understand the injury mechanism, but it is difficult to obtain for ethical reasons. Large animal models that have close anatomical similarities to humans could be used as surrogates. Piglet models have been previously used to study the extent of BP deficits following injury. 10 In the current study, we reported the biomechanical properties of the BP using a neonatal porcine model (piglets).

**Table 3:** Summary of existing literature reporting failure/rupture responses and mechanical responses from brachial plexus tissue

Author	Source	Mechanical findings		Loading rate
Kawai et al 12	Rabbit (fresh)	*Failure loads* : Upward: 20 N Lateral: 23 N Downward: 38 N *Stress* : Nerve rupture: 46 MPa Root avulsion: 26 MPa	*Strain at failure* : Nerve rupture: 7–9% Root avulsions: 7%	500 mm/min
Narakas 13	Human (patients)	70% nerve root avulsion (clinical finding)		
Marani et al 11	Human (fixed and unfixed)	*Failure stress* : Fixed nerves: 0.25 N/mm ^2^ Unfixed nerves: 0.14 N/mm ^2^	Strain at failure at high rate: Fixed nerves: 5% Unfixed nerve: 3.3%	10, 20, 50, 500 mm/min
Zapałowicz and Radek 14	Human (fresh)	Failure force: 217.7 N–546.3 N Failure stress: 1.3–3.5 N/mm ^2^	Strain at failure: 19.6–58.8%	
Kleinrensink et al 15	Human (fixed)	Force median nerve: 6.71 N Ulnar nerve: 4.80 N Radial nerve: 5.88 N (non-failure responses during upper limb tension test)		
Zapałowicz and Radek 16	Human (unknown)	Maximum force: 630 N	Strain at maximum force: 37%	20 cm/min

As shown in Table 3 , available studies on the tensile properties of BP are limited to responses at high stretch rates (10–200 mm/min). This study appears to be the first to demonstrate the mechanical behavior of BP at lower stretch rates (0.01 mm/second or 0.6 mm/min) and compare their responses to those at higher stretch rates (10 mm/second or 600 mm/min). Furthermore, this study provides the biomechanical properties of the anatomically complex BP structure, which consists of various segments including roots, trunks, chords, and BP nerves and compare their properties to another peripheral nerve, the tibial nerve. Most important finding from this study is that there is a significant effect of the stretch rate on the biomechanical responses of BP and that the BP segments vary in their responses.

Previous study by Marani et al tested fixed and unfixed BP at four different velocities (10, 20, 50, and 500 mm/min). 11 They reported only the effects of stretch rates on the failure length of the tissue. No information was provided on the effects of these different velocities on the load, stress, or E values. This study reported a significant effect of stretch rate with an increase in the maximum load, maximum stress, and E values at higher stretch rates. Knowing the effects of stretch rates on the BP helps better understand the NBPP-associated injury mechanisms as this information can be used to develop a biofidelic computational model that accurately illustrates the predisposing risk factors for BP injury.

Another study by Kawai et al reported the failure load and stress in rabbit BP when stretched in upward, downward, and lateral directions at 500 mm/min. 12 The site and level of injury was directly related to the direction of stretch. The maximum loads reported were 20 to 38 N when pulled in various directions. This study reported the BP nerve failure at 4.97 ± 1.84 N load at a 10 mm/second or 600 mm/min stretch rate. The observed lower load values in the current study can be attributed to the difference in the testing setup between the two studies. Current study excised the BP segments and the testing was performed in vitro. Kawai et al performed stretches in vivo while the BP segments were still intact in a euthanized animal. Thus, failure load values in Kawai et al's study were reported from the entire BP and not just the isolated segments of the BP. Additionally, neonatal animal model was used in the current study as compared with the adult animal used in Kawai et al study.

Although several studies have reported root avulsion injuries (Erb's palsy) to be most prevalent during NBPP, it is still unclear if the difference in the mechanical properties, including load, stresses, and E values, among various segments of the BP is due to the structural nonhomogeneity of the tissue. While it is known that the nerve tissue is a nonhomogeneous structure, there are no available data on the relative amount of axons, myelin, Schwann's cells, endoneurium, perineurium, epineurium, connective tissue, and blood vessels in the composition of various BP segments. Reported differences in the maximum stresses and E values induced at various BP segments and those in tibial nerve suggest differences in the tissue composition warranting future studies that aim to investigate structural compositions of the BP complex and other peripheral nerve, especially those that are used for surgical interventions while treating NBPP.

In this study, video data revealed differences in the segment failure patterns suggesting variation in the mechanical behavior of the segments and other nerves at different stretch rates. In case of the quasistatic stretch rate, necking in the sample at the proportional limit with an intact outer sheath/tissue suggests that the majority of force, before maximum load, might be sustained by some of its interior structures and that after these failure, the outer sheath/tissue continues to take load as shown at Point C in Fig. 5 . Occurrence of necking was significantly higher in the root/trunk (71%) followed by chord (22%), and BP nerve (14%), as well as tibial nerve (12%). When subjected to higher stretch rates, a sudden partial rupture of the sample suggests more uniform distribution of the load among the structural elements of the tissue during the pull. Occurrence of partial rupture was again significantly higher in the root/trunk (90%) followed by chord (46%) and then BP nerve (30%) segments as well as in the tibial nerve (32%). These variations in the failure patterns further warrant histological studies investigating anatomical outcomes of stretches in tested BP segments at specified strain levels and rates.

In summary, this study is the first to report biomechanical properties of neonatal BP in a piglet animal model at three different BP segments and two different stretch rates and to compare their properties to another peripheral nerve. The data obtained from this study can be used to develop a biofidelic computational model that accurately illustrates the predisposing risk factors for BP injury and help advance the science of obstetrical care.
